# Systemic Effects of mitoTEMPO upon Lipopolysaccharide Challenge Are Due to Its Antioxidant Part, While Local Effects in the Lung Are Due to Triphenylphosphonium

**DOI:** 10.3390/antiox11020323

**Published:** 2022-02-06

**Authors:** Adelheid Weidinger, Linda Birgisdóttir, Julia Schäffer, Andras T. Meszaros, Sergejs Zavadskis, Andrea Müllebner, Matthias Hecker, Johanna Catharina Duvigneau, Natascha Sommer, Andrey V. Kozlov

**Affiliations:** 1 Ludwig Boltzmann Institute for Traumatology, The Research Center in Cooperation with AUVA, 1200 Vienna, Austria; adelheid.weidinger@trauma.lbg.ac.at (A.W.); linda.birgisdottir@oulu.fi (L.B.); sergejs.zavadskis@trauma.lbg.ac.at (S.Z.); 2 Austrian Cluster for Tissue Regeneration, 1200 Vienna, Austria; 3 Faculty of Biochemistry and Molecular Medicine, University of Oulu, 90570 Oulu, Finland; 4 Department of Internal Medicine, Universities of Giessen and Marburg Lung Center (UGMLC), Member of the German Center for Lung Research (DZL), Excellence Cluster Cardio-Pulmonary Institute (CPI), Justus-Liebig University, 35392 Giessen, Germany; Julia.Schaeffer@innere.med.uni-giessen.de (J.S.); Matthias.hecker@innere.med.uni-giessen.de (M.H.); Natascha.Sommer@innere.med.uni-giessen.de (N.S.); 5 Department of Visceral, Transplant and Thoracic Surgery, Medical University of Innsbruck, 6020 Innsbruck, Austria; andras.meszaros@i-med.ac.at; 6 Department for Biomedical Sciences, Institute for Medical Biochemistry, University of Veterinary Medicine, 1210 Vienna, Austria; Andrea.Muellebner@vetmeduni.ac.at (A.M.); Catharina.Duvigneau@vetmeduni.ac.at (J.C.D.); 7 Laboratory of Navigational Redox Lipidomics, Department of Human Pathology, IM Seche-Nov Moscow State Medical University, 119146 Moscow, Russia

**Keywords:** mitochondria-targeted antioxidants, mitoTEMPO, TPP, lipopolysaccharide, inflammatory response, lung, liver, pulmonary artery pressure, edema

## Abstract

Mitochondria-targeted antioxidants (mtAOX) are a promising treatment strategy against reactive oxygen species-induced damage. Reports about harmful effects of mtAOX lead to the question of whether these could be caused by the carrier molecule triphenylphosphonium (TPP). The aim of this study was to investigate the biological effects of the mtAOX mitoTEMPO, and TPP in a rat model of systemic inflammatory response. The inflammatory response was induced by lipopolysaccharide (LPS) injection. We show that mitoTEMPO reduced expression of inducible nitric oxide synthase in the liver, lowered blood levels of tissue damage markers such as liver damage markers (aspartate aminotransferase and alanine aminotransferase), kidney damage markers (urea and creatinine), and the general organ damage marker, lactate dehydrogenase. In contrast, TPP slightly, but not significantly, increased the LPS-induced effects. Surprisingly, both mitoTEMPO and TPP reduced the wet/dry ratio in the lung after 24 h. In the isolated lung, both substances enhanced the increase in pulmonary arterial pressure induced by LPS observed within 3 h after LPS treatments but did not affect edema formation at this time. Our data suggest that beneficial effects of mitoTEMPO in organs are due to its antioxidant moiety (TEMPO), except for the lung where its effects are mediated by TPP.

## 1. Introduction

Understanding mechanisms that control intracellular signaling is an important but difficult task because signaling molecules, such as reactive oxygen species (ROS) are very short lived. Giant steps forward in understanding the role of mitochondrial ROS (mtROS) in physiological and pathological processes were made with the help of so-called mitochondria-targeted antioxidants (mtAOX). This class of molecules has two components. In the majority of cases, triphenylphosphonium (TPP) acts as the mitochondria-targeting moiety. TPP is a hydrophobic, positively charged molecule that selectively accumulates in mitochondria due to their unusually high membrane potential [1]. The second part of the molecule, the functional moiety comprising different types of antioxidants, is delivered to mitochondria and serves as a trap for ROS.

The effects of mtAOX, as described in the literature, are predominantly beneficial, mitigating pathological changes in the body [2,3,4,5,6]. However, there are also reports showing that the effect of mtAOX can be harmful [7]. The reason for this discrepancy is not clear, although recent data suggest that targeted antioxidants can act in different manners. Particularly, it has been shown that the carrier part of the molecule, in most cases TPP, can also manifest biological effects. For example, Powell and coauthors reported that intravenous injection of TPP in rats resulted in an increase in antioxidant activity in the liver, kidney, and spleen, but not in the lung [8]. In contrast, the antioxidant activity was decreased in the lung [8]. Interestingly, antioxidant activity did not correlate with levels of lipid peroxidation, which was increased in all organs [8]. Furthermore, it has been shown that different derivatives of TPP, which do not exert antioxidant properties, can substantially drop the rate of mitochondrial respiration linked to the major mitochondrial function of ATP synthesis [9]. It has also been shown that TPP molecules induce a significant increase in proton leak with a decrease in the mitochondrial membrane potential and an inhibition of respiratory chain complexes [10].

Indeed, it is known that a drop in mitochondrial membrane potential can be an additional mechanism that decreases the generation of mitochondrial ROS. For example, up-regulation of mitochondrial uncoupling proteins or treatment with ionophors such as carbonylcyanide-p-tri-fluoromethoxyphenylhydrazon can decrease the generation of mitochondrial ROS [11] and reduce ROS-mediated oxidative damage as well as ROS-mediated intracellular signaling [12]. Thus, theoretically, both an mtAOX such as mitoTEMPO, as well as its carrier molecule TPP alone, could reduce ROS production, either indirectly by decreasing mitochondrial membrane potential or directly by scavenging ROS.

There is a body of literature providing evidence for the involvement of ROS in the pathogenesis of pulmonary inflammation [13,14,15]. The most common way to induce an inflammatory response is the treatment with lipopolysaccharide (LPS). It has been shown that LPS stimulates ROS production in lung macrophages and endothelial cells [16] and that ROS increase endothelial permeability in the lung [16,17,18]. It has also been shown that pre-treatment with mitoTEMPO, a scavenger of mtROS, attenuated LPS-induced ROS generation and decreased lung endothelial permeability in an in vitro model [18].

The aim of this study was to compare the biological effects of TPP and mitoTEMPO in different organs in a rat model of the systemic inflammatory response syndrome (SIRS) induced by endotoxin in order to clarify which part of mitoTEMPO, TPP, or TEMPO is responsible for its effects.

## 2. Materials and Methods

### 2.1. Chemicals

All reagents were obtained from Merck/Sigma-Aldrich (Vienna, Austria) unless otherwise noted.

### 2.2. Animals

The experiments were performed on male Sprague-Dawley rats (weighing 350–400 g; *n* is indicated in each figure legend; Charles River, Germany; Janvier Labs, France) which were kept under controlled standard animal housing conditions. The animals had free access to standard laboratory rodent food and water. They were kept for 7 days prior to usage in experiments for accommodation.

### 2.3. Lipopolysaccharide Treatment

In the experiments described here, we used two lots of LPS from *Escherichia coli* serotype 026:B6 (activity ≥500 000 EU/mg); each was tested for its effect on the survival rate. The dose corresponding to a survival rate slightly above 50% was selected (data not shown), which was 2.5 mg and 1.75 mg per kg body weight, respectively. LPS was dissolved in saline (Fresenius Kabi, Austria), and the LPS solution was vortexed for 1 min and sonicated for 30 s before application. Subsequently, the suspension was injected into the penis vein under isoflurane anesthesia in a volume ranging from 0.5 to 0.75 mL. Normal saline was used as a control. For analgesia, Buprenorphine (Richter Pharma AG, Wels, Austria, 0.05 mg/kg body weight) was injected subcutaneously at the time of the LPS treatment and 8–10 h thereafter. For in vivo treatment, methyltriphenyl-phosphonium chloride (TPP, 50 nmol/kg) or mitoTEMPO (50 nmol/kg) was injected intraperitoneally one hour before the LPS/saline injection.

### 2.4. Blood and Organ Sampling for Nitrite, Nitrogen Oxides, Organ Damage Marker and Gene Expression Measurements

The experimental endpoint was 16 h after LPS/saline injection. Blood was collected in a lithium heparin tube from the tail vein before treatment and from the heart at 16 h. Heparin tubes were centrifuged at 400× *g* for 10–15 min and plasma was collected. The rats were euthanized by injection of 2 mL thiopental-natrium and sodium carbonate (Thiopental, Rotexmedica GmbH, Germany) under anesthesia, and organs were collected immediately into ice-cold Ringer solution (Fresenius Kabi, Germany), cut into small pieces, transferred to liquid nitrogen, and stored at −80 °C until further measurement.

### 2.5. Animal Experiments, Sampling of Blood, Peritoneal and Bronchial Lavage for H_2_O_2_ Measurement

**Figure 1:**
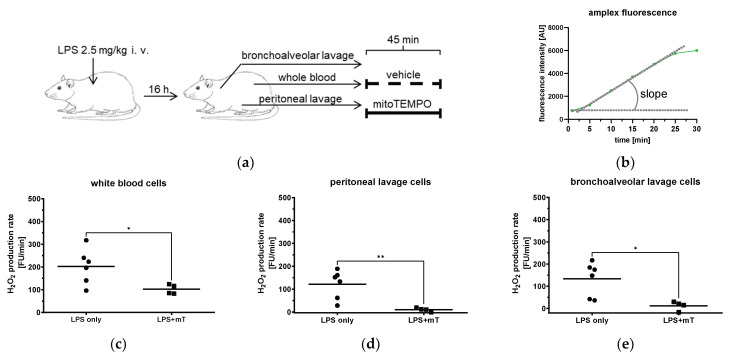
Effect of mitoTEMPO (mT) on generation of ROS in different body compartments of rats. (**a**) Rats were challenged with 2.5 mg/kg body weight lipopolysaccharide (LPS) and biological fluids were withdrawn after 16 h. (**b**) Scheme of H_2_O_2_ production rate assessed by Amplex Red by calculating the slope. (**c**–**e**) Effect of mitoTEMPO on H_2_O_2_ production rate of white blood cells (**c**), peritoneal lavage (**d**), and bronchoalveolar lavage (**e**). Unpaired *t*-test, * *p* < 0.05, ** *p* < 0.01.

The experimental model used for the measurement of H_2_O_2_ in different body compartments is illustrated in Figure 1a. At the end of the LPS treatment, rats were anesthetized by inhalation of a mixture of 3% isoflurane and oxygen. After a small skin cut, the left femoral artery was dissected and catheterized using a 24-gauge i.v. cannula (BD Neoflon, Becton Dickinson Infusion Therapy AB, Helsingborg, Sweden). Ten milliliters of whole blood was collected in a 50 mL Falcon tube prefilled with 200 µL of sodium heparin (1000 IU/mL, Gilvasan Pharma GmbH, Vienna, Austria) for subsequent processing and for the in vitro part of the study. Following the blood sampling, the animals were euthanized by decapitation.

For bronchoalveolar lavage, we used a protocol adapted from [19]. Briefly, a 19-gauge needle hub was inserted into the trachea and 3 mL of ice-cold phosphate-buffered saline (PBS) and 10% fetal bovine serum (FBS) were administered and aspirated slowly through the needle hub. The fluid was then transferred into a 15 mL tube (Greiner Bio-One, Austria) and kept on ice. The procedure was repeated four times.

To collect peritoneal cells, the peritoneum was exposed, and 5 mL of ice-cold PBS with 3% FBS was injected into the peritoneal cavity through a needle, as described elsewhere [20]. A gentle massage to the abdomen was applied to dislodge any attached cells into the PBS solution. Then, the suspension was collected through a 22-gauge needle into a syringe and transferred into a 15 mL tube and kept on ice. The above-described procedure was repeated five times.

#### 2.5.1. Purification of Cells and Incubation with mitoTEMPO

The suspension of bronchoalveolar and peritoneal cells was centrifuged at 400× *g* for 10 min at 4 °C, the supernatant was discarded, and the cell number was counted with a Cell-Dyn 3700 Hematology Analyzer (Abbott Laboratories, Lake Bluff, IL, USA). After a complete blood count, blood samples were centrifuged at 300× *g* for 10 min at 4 °C, then the plasma and buffy coat were removed and the remaining blood pellet was incubated with a lysis buffer containing 168 mM NH_4_Cl, 10 mM KHCO_3_, and 973 µM EDTA for 10 min at 4 °C. Cells were then washed twice with PBS at 10 °C followed by centrifugation at 400× *g* for 8 min and finally, counts were performed. For the in vitro mtAOX treatment, cell suspensions were incubated at 37 °C for 45 min with mitoTEMPO (500 nM) or with vehicle (NaCl).

#### 2.5.2. Extracellular H_2_O_2_ Measurement

The H_2_O_2_ production in the cell suspension was assessed by N-acetyl-3,7-dihydroxyphenoxazine (Amplex Red, Life Technologies, Eugene, OR, USA). Amplex Red is a sensitive and chemically stable fluorogenic probe and produces the fluorescent resolufin with H_2_O_2_ in a horseradish peroxidase (HRP)-catalyzed oxidation, with excitation/emission maxima at 563/587. Reaction stoichiometry of Amplex Red and H_2_O_2_ is 1:1 [21]. Using a fluorescence plate reader (POLARstar Omega 3MG, Labtech, Germany), white blood cells, bronchoalveolar lavage, and peritoneal lavage cells (15 × 10^3^ cells per well in Krebs buffer) were incubated in black 96-well microplates (Greiner Cellstar) at 37 °C with Amplex Red (10 µM) and HRP (0.2 U mL^−^^1^). The fluorescence intensity was recorded for 30 min with an excitation of 544 nm/emission of 590 nm and gain at 1200. The slope was calculated from a 10 min interval between the 5th and 15th min of the measurement nm (Figure 1b).

### 2.6. Nitrite and Nitrogen Oxides Measurement

Nitric oxide (NO) was measured in duplicates from plasma samples with the Sievers 280i-NO Analyzer (General Electric’s Analytical Instruments, Boulder, USA). Prior to measuring, a calibration curve was recorded using a standard stock solution (0.069 g NaNO_2_ in 10 mL dH_2_O). For nitrogen oxides (NOx) measurement, vanadium (III) chloride was applied (0.2 g V(III)Cl_3_ in 25 mL of 1 M hydrochloride acid (HCl)) as a reactive agent in a 95 °C water bath (Grant Instruments, Beaver Falls, USA). For nitrite (NO_2_^−^) measurement, 50–100 µL sodium iodide (0.05 g NaI in 0.5 mL dH_2_O supplemented with 5 mL acetic acid) was applied as a reactive agent.

### 2.7. Determination of Heme Oxygenase Activity

Liver tissue was homogenized using a Potter-Elvehjem with PTFE pestle on ice in 1:10 weight to volume ratio in a Tris buffer (20 mM) containing 300 mM sucrose and 8 µM EDTA (pH 7.4). Samples were distributed in aliquots of 300 µL and snap frozen in liquid nitrogen until activity measurement. The residual homogenate was used to constitute a pool, which was used as an internal standard (IS). For the determination of HO activity, a coupled enzyme assay was used, which determines the end-product bilirubin (BR). For each sample, which was analyzed in duplicates, 25 µL of homogenate was added to ice-cold 92 µL HO-assay buffer (KH_2_PO_4_ (25 mM) and K_2_HPO_4_ (75 mM) (pH 7.4), 8 µL deferoxamine (DFO, 100 mM, Desferal^®^, Novartis, Switzerland), 4 µL hemin (5 mM, Fluka, Buchs, Switzerland), supplemented with 25 µL NADPH (10 mM, Sigma). For each sample, one control was included containing 4 µL DMSO instead of hemin and 25 µL HO-assay buffer instead of NADPH. Samples were vortexed and incubated for 30 min at 37 °C under constant agitation. The reaction was stopped by transferring the samples on ice. BR was extracted into benzene as described previously [22]. BR concentration in extracts was determined using a double beam spectrophotometer (U-3900, Hitachi, Tokyo, Japan) and a BR standard calibration curve. The detection limit of BR using this method was determined as 5 pmol BR. We found that the biliverdin reductase activity (the capacity to convert biliverdin into BR) in liver tissue was much higher (<10 times, data not shown) than that of HO, indicating that BVR activity is not limiting and BV formed by the HO enzyme is completely reduced to BR by the underlying BVR. The amount of BR determined in samples was corrected for the BR amount determined in the negative controls and corrected for the underlying protein content. Protein determination was performed using the Bradford method and a BSA calibration curve, as described earlier [22]. Enzyme activities were expressed as nmol BR formed per mg protein in 30 min.

### 2.8. Gene Expression of Inflammatory and Cell Stress Markers, iNOS, HO-1, IL6, TNFR1, NFkBia and CHOP

Gene expression analysis was performed using qPCR as described elsewhere [23]. RNA was isolated from 100 µL liver homogenate prepared as described above using 1 mL of TriReagent^TM^ (MRC, Cincinnati, OH, USA). Extraction of RNA was performed according to the manufacturer’s protocol. The amount of extracted RNA was determined spectrophotometrically at 260 nm and purity was assessed by the 260/280 nm ratio on an Eppendorf BioPhotometer plusUV/VIS (Eppendorf, Hamburg, Germany). Copy DNA was prepared as previously described [23]. Equal aliquots from each cDNA were pooled to generate an internal standard (IS) which was used as a reference for the quantification. Primer pairs used for the expression analysis of targets and the internal reference genes, hypoxanthine ribosyltransferase (HPRT), and cyclophilin A (Cyc) are described in detail in Table A1. PCR reactions contained iTaq™ DNA polymerase™ (0.625 U/reaction; BioRad), primers (250 nmol/L each, Invitrogen), dNTP at a final concentration of 200 µmol/L (each), 3 mmol/L MgCl_2_ (except for IL-6, 2 mmol/L; iNOS, 1.5 mmol/L), and SYBR^®^ green I as reporter dye (0.5×) in the provided reaction buffer at a final volume of 12 µL. The qPCR was carried out on a CFX96^TM^ (Bio-Rad, Hercules, CA, USA) real-time PCR detection system. Data were analyzed using the inbuilt software CFX manager (Version 2.0, Bio-Rad) in the linear regression mode. A modified comparative ΔΔCq method [24] was applied for data analyses. All data were normalized for the reference genes Cyclophilin A and hypoxanthinribosyl transferase (HPRT) and calculated as fold change relative to the IS.

### 2.9. Organ Damage Markers

Plasma levels of aspartate aminotransferase (AST), alanine aminotransferase (ALT), lactate dehydrogenase (LDH), urea, creatinine (crea), and creatinine kinase (CK) were analyzed with the Roche Cobas cIII analyzer (Roche, Basel, Switzerland) according to the manufacturer’s instruction.

### 2.10. Wet/Dry Ratio

Approximately 0.2–0.3 g of lung tissue was used to determine the wet/dry ratio. The tissue was weighted before and after freeze drying (Christ Alpha 1-2 LD plus, Osterode am Harz, Germany). A program of −49.6 °C and vacuum of 0.63 mbar was operated for approximately 18 h. The ratio between wet and dry weight of the lung tissue was calculated.

### 2.11. Isolated Perfused and Ventilated Lungs

Isolated perfused and ventilated lungs were prepared as previously described [25]. Briefly, male C57BL/6J mice were deeply anesthetized by an intraperitoneal injection of ketamine (100 mg/kg bodyweight (BW)) and xylazine (20 mg/kg BW) with heparin (50,000 I.U./kg BW) as an anticoagulant. Mice were intubated via a tracheostoma and ventilated with normoxic gas (21% O_2_, positive pressure ventilation, 10 µL/g BW tidal volume, 90 breath/min, and 3 cm H_2_O positive end-expiratory pressure). After opening the chest, catheters were inserted in the pulmonary artery and the left atrium and perfused with Krebs–Henseleit buffer (Serag Wiessner, Naila, Germany) using a peristaltic pump (ISM834A V2.10, Ismatec) to reach a final flow of 2 mL/min. In parallel with the onset of artificial perfusion, ventilation was changed from ambient air to a pre-mixed gas (21% O_2_, 5.3% CO_2_, balanced with N_2_). The lungs were removed from the thorax and freely suspended on a force transducer to monitor organ weight. After reaching a stable state, 6 µg/mL (final concentration) of lipopolysaccharide (LPS, Samonella enterica serotype abortus equi) was added to the perfusate and recirculated for 2 h, followed by application of mitoTEMPO/TPP or the solvent as control each 10 min (minute: 145, 155, 165, 175) in increasing concentrations (final concentrations: 0.1, 0.5, 1.0, 10 µM). The pulmonary arterial pressure (PAP) and lung weight (LW) were monitored continuously during the experiment. Changes in PAP (ΔPAP) and lung weight (ΔLW) were determined by calculating the difference in the respective value averaged from minute 9 to 10 after mitoTEMPO, TPP, or solvent application and the value averaged over one minute before mitoTEMPO, TPP, or solvent application.

### 2.12. Statistical Analysis

Data were analyzed using GraphPad-Prism software (8.0.1.244, USA) by unpaired *t*-test, one-way analysis of variance (ANOVA) followed by Fisher’s least significant difference test, or by two-way ANOVA with Tukey’s post hoc test. In all tests, *n* (sample size) represents biological replicates (donors). The statistical tests and the *n* number are indicated in the figure legends. Results are presented as the mean ± SEM. Level of significance was set at 0.05 and is indicated as * *p* < 0.05, ** *p* < 0.01, *** *p* < 0.001, **** *p* < 0.0001.

## 3. Results

Immune cells, particularly macrophages and monocytes, are the first components of the innate immune system responding to bacterial toxins by the elevated generation of ROS. Therefore, we first tested whether mitoTEMPO is able to inhibit ROS generation in different body compartments in our model. We analyzed the ROS (H_2_O_2_) production capacity of immune cells derived from three compartments, namely blood (white blood cells (WBC) isolated from the blood), peritoneal lavage, and bronchoalveolar lavage from animals treated with LPS in vivo (Figure 1a). To avoid a complex procedure of normalization, we tested the response of the cells within the biological compartments to mitoTEMPO ex vivo (Figure 1a). The corresponding cell preparations were divided into two aliquots; one was incubated with saline, the other one with mitoTEMPO. The results show that mitoTEMPO efficiently decreases ROS generation in cells from all three compartments (Figure 1c–e), indicating that mitochondrial ROS-mediated pathways are operating in our model. Since not only macrophages but also other lung cells may generate ROS, we also confirmed these findings in precision-cut lung slices (PCLS, Supplementary Materials S1). PCLS contain all lung cell types and maintain tissue structure but are not influenced by systemic factors and are therefore suitable to investigate local effects in the tissue. We showed that incubation with LPS increases cytoplasmic ROS and both TPP and mitoTEMPO attenuate ROS levels (Supplementary Figure S1), suggesting that a part of cytoplasmic ROS is released from mitochondria. In addition, ROS from non-mitochondrial sources could also play a role, which could be attenuated by non-targeted antioxidant TEMPO [26]. In our study, we did not consider TEMPO as an appropriate control, because, as mentioned, it accumulates in all cell compartments and can exert non-mitochondrial effects. Since both TPP and mitoTEMPO accumulate and act in mitochondria, we studied separately effects of both these molecules in the following experiments.

**Figure 2:**
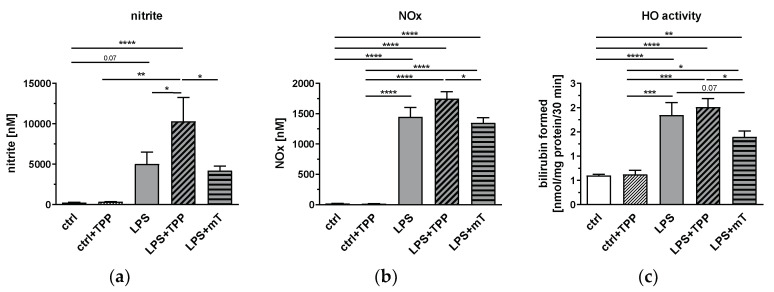
Effect of lipopolysaccharide (LPS, 1.75 mg/kg), methyltriphenylphosphonium chloride (TPP, 50 nmol/kg) and mitoTEMPO (mT, 50 nmol/kg) on the levels of nitric oxide (NO) derivatives (**a**), and nitrite and nitrogen oxide (NOx) in rat plasma (**b**), as well as heme oxygenase (HO) activity in liver tissue (**c**). MitoTEMPO decreases the activity of the tested metabolites (**a**–**c**) while TPP increases the mean values of NO derivatives (**a**) and NOx (**b**) in the blood. One-way analysis of variance (ANOVA), Fisher’s least significant difference (LSD), *n* = 3–8, mean ± SEM, * *p* < 0.05, ** *p* < 0.01, *** *p* < 0.001, **** *p* < 0.0001.

One of the characteristic features of the inflammatory response in the endotoxic shock model is the upregulation of inducible nitric oxide synthase (iNOS) and excessive formation of nitric oxide (NO). Therefore, we investigated the effects of TPP and mitoTEMPO on the levels of LPS-induced NO metabolites in blood plasma. As carbon monoxide generated by heme oxygenase (HO) could, similarly to NO, exert biological activities on mitochondria, we also determined HO activity in liver tissue. As expected, HO activity showed similar kinetics to all NO derivatives (Figure 2). Both nitrite and NOx as well as HO activity were elevated upon LPS treatment, and significantly attenuated by mitoTEMPO compared to the LPS + TPP group (Figure 2a–c). Interestingly, TPP slightly increased the mean values of all these parameters, but this increase was significant only for the plasma levels of nitrite. These data indicate that the beneficial effects of mitoTEMPO can only be achieved by its antioxidant moiety.

**Figure 3:**
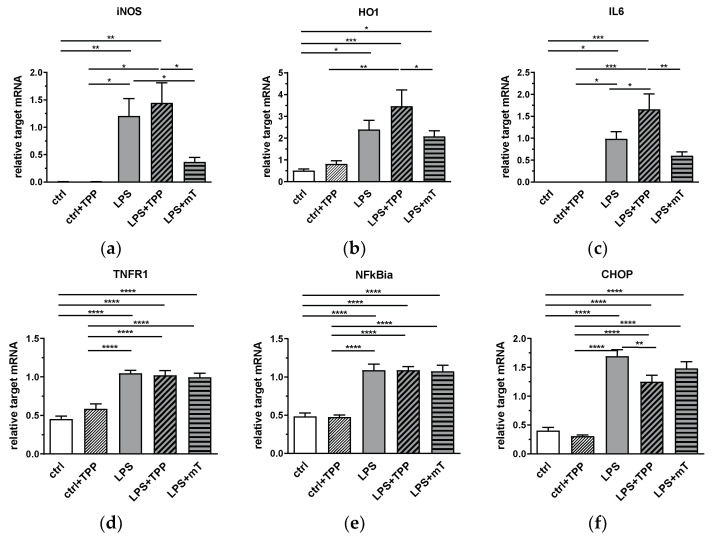
Effect of lipopolysaccharide (LPS, 1.75 mg/kg), methyltriphenylphosphonium chloride (TPP, 50 nmol/kg) and mitoTEMPO (mT, 50 nmol/kg) on gene expression of iNOS (**a**), HO1 (**b**), IL-6 (**c**), TNFR1 (**d**), NFkBia (**e**), and CHOP (**f**) in rat liver. After LPS injection, gene expression levels of all mediators increased (**a**–**f**). MitoTEMPO treatment decreased the mean values of iNOS (**a**) and IL-6 (**c**). An increase is seen in IL-6 expression in the LPS+TPP group compared to the other LPS groups. All data are normalized for the expression levels of the reference genes (cyclophilin A and hypoxanthine-guanine phosphoribosyl transferase (HPRT)) and expressed as fold changes relative to the internal standard. * *p* < 0.05, ** *p* < 0.01, *** *p* < 0.001. One-way analysis of variance (ANOVA), Fisher’s least significant difference (LSD), *n* = 3–8, mean ± SEM, * *p* < 0.05, ** *p* < 0.01, *** *p* < 0.001, **** *p* < 0.0001. Abbreviations: inducible nitric oxide synthase (iNOS), heme oxygenase 1 (HO-1), interleukin 6 (IL-6), tumor necrosis factor receptor 1 (TNF-R1), nuclear factor kappa B inhibitor alpha (NFkBia), CCAAT/enhancer-binding protein homologous protein (CHOP).

The liver plays an important physiological role in LPS detoxification and, in particular, hepatocytes are involved in the clearance of endotoxin of intestinal derivation [27]. Therefore, we measured the effect of mitoTEMPO on the inflammatory response in the liver. We determined the mRNA expression levels of iNOS and HO-1, as well as other inflammation-associated response genes, such as interleukin (IL)6, tumor necrosis factor receptor (TNFR)1, nuclear factor kappa B inhibitor alpha (NFkBia), and CCAAT/enhancer-binding protein homologous protein (CHOP) (Figure 3). We observed a clear response of all markers to the LPS challenge; their expression levels were increased (Figure 3). The treatment with mitoTEMPO decreased the mean values of iNOS and IL-6 (Figure 3a,c), whereas the treatment with TPP even further increased the gene expression levels, with that of IL-6 being significant. These data are in line with the previously reported data on beneficial effects of mtAOX such as mitoTEMPO [2,3,4,5,6]. Interestingly, we found that the LPS-mediated increase in the expression levels of TNFR1, NFkBialpha, and CHOP was not affected by mitoTEMPO, indicating that mainly the pathogen-associated molecules drive these targets. Except for CHOP, we found no down-modulating effect of TPP on the expression of target genes in the liver.

**Figure 4:**
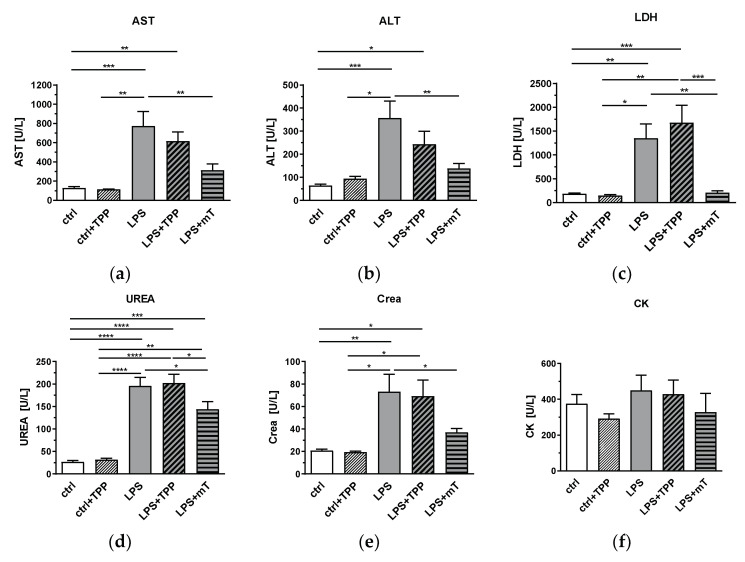
Effect of lipopolysaccharide (LPS, 1.75 mg/kg), methyltriphenylphosphonium chloride (TPP, 50 nmol/kg) and mitoTEMPO (mT, 50 nmol/kg) on organ damage markers. An increase for few values is seen in the LPS + TPP group compared to the LPS group for all tissue damage markers. LDH showed significant difference between the LPS + TPP and all other groups. The LPS + mitoTEMPO seems to indicate beneficial effects of antioxidants (AOX) being lower than both the LPS and the LPS + TPP groups for all values measured; (**a**) aspartate aminotransferase (AST), (**b**) alanine aminotransferase (ALT), (**c**) lactate dehydrogenase (LDH), (**d**) urea, (**e**) creatinine (Crea) and (**f**) creatine kinase (CK). One-way analysis of variance (ANOVA), Fisher’s least significant difference (LSD), *n* = 3–8, mean ± SEM, * *p* < 0.05, ** *p* < 0.01, *** *p* < 0.001, **** *p* < 0.0001.

Next, we investigated the effect of mitoTEMPO on tissue damage markers. MitoTEMPO significantly decreased the levels of the liver damage markers AST (Figure 4a) and ALT (Figure 4b), the organ damage marker LDH (Figure 4c), and the markers for kidney dysfunction and damage, urea (Figure 4d) and creatinine (Figure 4e) in plasma. All these markers showed a similar response, as we found for the expression of iNOS, HO-1, and IL-6 in the liver. Accordingly, TPP presence also showed no beneficial effect on these markers (Figure 4a–f). Our data indicate that mitochondrial ROS contribute, to a significant extent, to the LPS-triggered increase in the levels of liver and kidney damage markers and the hepatic expression of iNOS, HO-1, and IL-6, since the antioxidant moiety of mitoTEMPO was capable of counteracting their rise.

**Figure 5:**
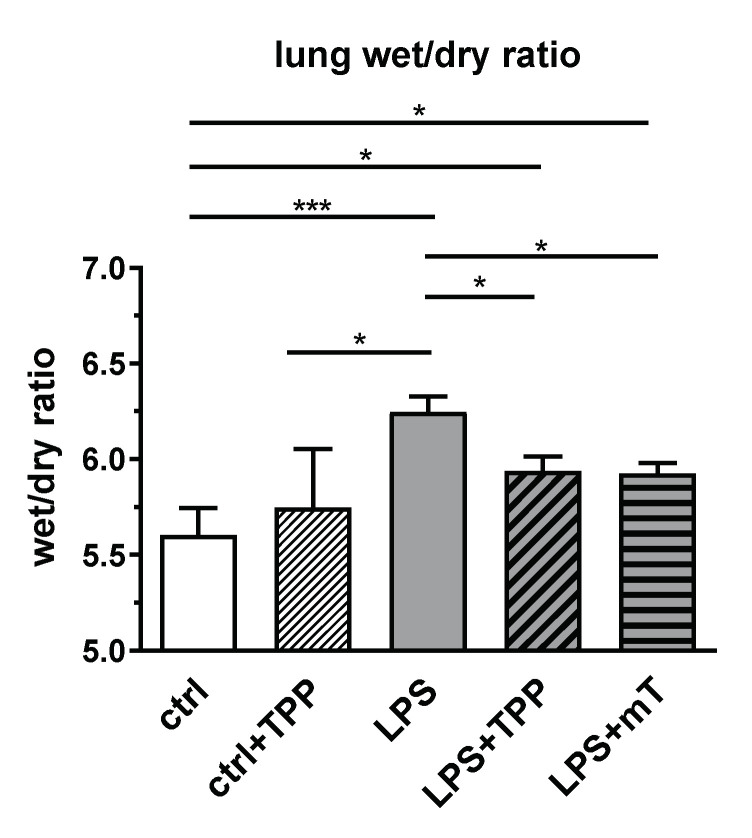
Effect of lipopolysaccharide (LPS, 1.75 mg/kg), methyltriphenylphosphonium chloride (TPP, 50 nmol/kg) and mitoTEMPO (mT, 50 nmol/kg) on the wet/dry ratio in rat lung. Treatment with lipopolysaccharide (LPS) elevated the wet/dry ratio whereas both TPP and mitoTEMPO reduced the wet/dry ratio to a similar extent. One-way analysis of variance (ANOVA), Fisher’s least significant difference (LSD), *n* = 3–8, mean ± SEM, * *p* < 0.05, *** *p* < 0.001.

Surprisingly, we observed a different picture in the lung. To estimate LPS-mediated damage, we determined the wet/dry ratio (W/D ratio) of lung tissue. LPS substantially elevated the W/D ratio, reflecting lung edema and serious lung damage (Figure 5). The application of mitoTEMPO and TPP showed that both compounds reduced the W/D ratio to a similar extent (Figure 5). This phenomenon led us to the assumption that it is the carrier molecule TPP that is responsible for the reduction of the W/D ratio in the lung and not the antioxidant part of mitoTEMPO.

**Figure 6:**
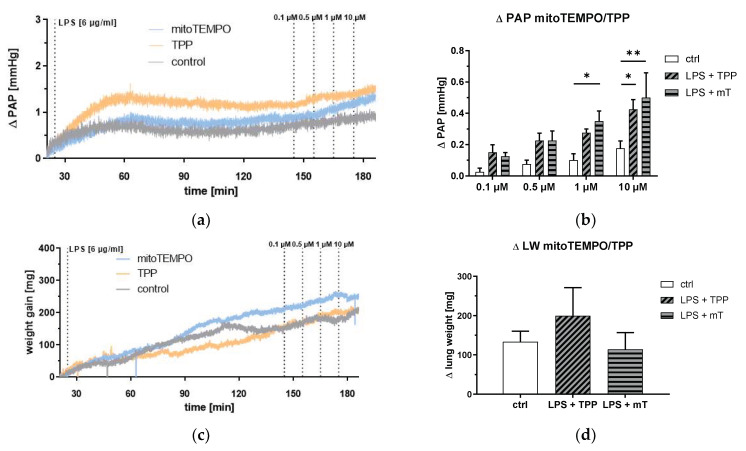
Isolated blood free perfused lung. Pulmonary arterial pressure (PAP) and lung weight (LW) in response to lipopolysaccharide (LPS, 6 µg/mL), methyltriphenylphosphonium chloride (TPP), and mitoTEMPO (mT) treatment. Course of PAP increase (**a**) or weight gain (**c**) after the start of the experiment (mean values of n = 4 isolated lungs each experiment). PAP increase (**b**) after repetitive application of mitoTEMPO/TPP and weight gain (**d**) after the last application of mitoTEMPO/TPP. Two-way analysis of variance (ANOVA) with Tukey’s post hoc test, *n* = 4 each group, mean ± SEM, * *p* < 0.05, ** *p* < 0.01.

This led us to a more profound investigation of mitoTEMPO/TPP action in the lung. To better dissect the effects of mitoTEMPO and TPP, we performed experiments in isolated lungs (ex vivo). Surprisingly, both mitoTEMPO and TPP strongly elevated PAP in LPS-treated isolated lungs, and no difference was observed between the effects of mitoTEMPO and TPP (Figure 6a,b). However, edema formation was not affected by the application of mitoTEMPO or TPP (Figure 6c,d). These findings support the notion that mitoTEMPO and TPP similarly affect the lung and that the increase in PAP and the development of edema have different mitoTEMPO/TPP-mediated regulatory mechanisms.

## 4. Discussion

Here, we show that the effects of TPP and mitoTEMPO differ in the lung from those elicited in the other organs. We observed that markers of organ damage as well as markers of the inflammatory response, particularly those associated with NO metabolism that increase in circulating blood, can be significantly lowered by mitoTEMPO, but not by TPP, pointing towards the role of ROS in the upregulation of theses markers in response to LPS. The effect of mitoTEMPO is in line with previous reports published by us and others in similar models of acute inflammation [2,3,4,5,6]. In contrast, both TPP and mitoTEMPO reduced the W/D ratio of the lung 24 h after LPS treatment to a similar extent. Moreover, both similarly elevated pulmonary arterial pressure (PAP) in isolated lung within 3 h after LPS treatment. The elevated PAP determined in isolated lung, however, was not accompanied by enhanced pulmonary edema formation. In general, this discrepancy between data obtained in the lung and other organs suggests that the TPP part is responsible for the effects in the lung and the TEMPO part for the effects in the rest of the body determined at the later time points. We do not know exactly how TPP acts in the lung, but there are several explanations based on the published data.

A single injection of LPS almost immediately elevates PAP, which drops after a couple of hours [28]. Lung edema in rats subjected to LPS starts at 6–8 h, reaching a maximum by 24 h [29]. These observations support the assumption that there are different mechanisms regulating PAP and edema in the endotoxic shock model.

The increase in PAP in response to LPS has been associated with the release of vasoconstrictors [28]. This is a result of the direct interaction of LPS with endothelial cells via toll-like receptor (TLR) 4 which activates the release of intracellular ROS [30]. A number of vasoactive substances are potentially capable of regulating PAP. One of the most prominent is endothelin 1 (ET-1), which is released by endothelial cells and regulated by ROS. ET-1 is activated by the endothelin-converting enzyme (ECE). This activation is regulated by ROS; superoxide radicals (O_2_^•−^) can inhibit endothelin-converting enzyme activity (ECE) and decrease ET-1 synthesis [31]. Thus, scavenging of O_2_^•−^ can activate ECE and increase PAP. This is in line with the effect of mitoTEMPO/TPP on PAP, if we consider that the reduction in membrane potential has a stronger impact on lowering the ROS level than scavenging of ROS. This assumption is supported by our recent observation on inhibition of ROS formation by TPP in pulmonary smooth muscle cells [32]. The development of LPS-induced edema is very often associated with elevated PAP. In our experiments, both mitoTEMPO and TPP decreased the W/D ratio in vivo and did not affect weight gain in the isolated lung, but elevated PAP. We assume that the development of lung edema and the increase in PAP in the isolated lung are regulated by different mechanisms. First, they occur at different time points. In response to LPS, we observed a continuous increase in weight in the isolated lung, while the PAP increased strongly at the very beginning of LPS treatment. Second, mitoTEMPO/TPP exerted different effects on PAP and weight gain.

In addition, although the mechanisms increasing PAP and inducing edema are different, they have a common feature being activated by ROS. We assumed that similar effects of mitoTEMPO and TPP occurred in the lung, because both modulate ROS production in a similar manner. Since increased ROS levels in response to LPS can originate not only from macrophages but also from epithelial cells, we examined this assumption in PCLS by means of laser scanning microscopy. PCLS comprise all types of lung cells and therefore, we expected that we can test our assumption in this experimental model. Indeed, as expected, we observed a drastic increase in ROS levels in PCLS in response to LPS and both TPP and mitoTEMPO abolished this effect. This supports our assumption that ROS are involved in the regulation of PAP and edema at early and late phases of the inflammatory response, respectively.

Indeed, ROS affect alveolar amiloride-sensitive epithelial sodium channels (ENaCs), which play a crucial role in sodium transport and fluid reabsorption in the lung [33]. LPS, frequently employed to induce acute liver injury in experimental animal models, has been reported to regulate ENaC expression and alveolar fluid clearance. ENaC has a critical role in the clearance of edema fluid from the alveolar space. Several lines of evidence suggest that protein kinase C (PKC) is likely to play a permissive role in hydrogen peroxide (H_2_O_2_)-mediated endothelial permeability and lung edema [34,35]. It seems that ROS released by macrophages are involved in edema induction. Reduction of endotoxin-induced macrophage activation results in the reduction of lung edema and mortality in vivo [36]. Furthermore, it has even been shown that H_2_O_2_ may enter the mesenteric lymph and travel to the lung, where it induces edema [37]. All these data suggest that the development of pulmonary edema in vivo is largely affected by systemic activation of the immune system, in addition to local pulmonary effects, which may explain the different effects of mitoTEMPO/TPP on edema formation in vivo and in the isolated lung. Inhibition of ROS generation in macrophages may mitigate epithelium damage and prevent edema formation in vivo but not ex vivo. Moreover, time-dependent effects may play a role, as in the isolated lung, edema formation was determined 150 min after LPS application (in vivo after 24 h). MitoTEMPO was applied in the isolated lung in a therapeutic manner 120 min after LPS application when edema formation was already triggered. This suggests that both ROS originating from circulating immune cells and ROS formed in lung tissue contribute to the induction of edema. In contrast, an increase in PAP is a result of interaction between endothelial cells and LPS. Thus, we assume that there are two separate mechanisms mediated by ROS, causing either increased PAP or the development of edema. This assumption adequately explains the action of reduced ROS on PAP and edema. Further studies are required to understand the interaction between mitochondria-targeted antioxidants and lung tissue in detail.

Conclusively, our findings suggest a different role of lung mitochondria for the development of the pathological effects associated with systemic inflammation, compared to the other organs. As TPP is sufficient for mitigating those effects, it may become a useful therapeutic intervention that specifically targets the lung.

## Data Availability

Data are contained within the article and supplementary material.

## Supplementary Materials

The following supporting information can be downloaded at: https://www.mdpi.com/article/10.3390/antiox11020323/s1, Supplementary Materials S1: Preparation of lung tissue slices, Figure S1: LSM images of precision cut lung slices.

## Appendix A

**Table A1:** Sequences for primers used for measuring gene expression. HPRT: hypoxanthine-guanine phosphoribosyl transferase; CHOP: CCAAT/enhancer-binding protein homologous protein; IL6: interleukin 6; iNOS: inducible nitric oxide synthase; HO1: heme oxygenase 1; NFkBia: NFkB inhibitor alpha; TNFR1: tumor necrosis factor receptor 1.

Gene	Accession Number	Primer Sequence	Source
Cyclophilin A	M19533	F: TAT CTG CAC TGC CAA GAC TGA GTG R: CTT CTT GCT GGT CTT GCC ATT CC	[24]
HPRT	NM_012583	F: CTC ATG GAC TGA TTA TGG ACA GGA C R: GCA GGT CAG CAA AGA ACT TAT AGC C	[24]
CHOP	NM_024134.2	F: TTG GGG GCA CCT ATA TCT CA R: CTC CTT CAG TCG CTG TTT CC	[24]
IL6	NM_012589.1	F: CCG GAG AGG AGA CTT CAC AG R: ACA GTG CAT CAT CGC TGT TC	[23]
iNOS	NM_012611.3	F: AGG CAA GCC CTC ACC TAC TT R: GTG GGG TTG TTG CTG AAC TT	[23]
HO1	NM_012580.2	F: CCA GCC ACA CAG CAC TAC R: GCG GTC TTA GCC TCT TCT G	[24]
NFkBia	NM_001105720.2	F: GCA ACA GCT CAC GGA GGA CGG R: TCC ACG ATG CCC AGG TAG CCA	Desinged for this study
TNFR1	NM_013091.1	F: GTG CCA CAA AGG AAC CTA CTT G R: AGG CTG GAG TTA GGG GCT TA	[38]

## References

[1] Skulachev V.P. (2005). How to clean the dirtiest place in the cell: Cationic antioxidants as intramitochondrial ROS scavengers. IUBMB Life.

[2] Patil N.K., Parajuli N., MacMillan-Crow L.A., Mayeux P.R. (2014). Inactivation of renal mitochondrial respiratory complexes and manganese superoxide dismutase during sepsis: Mitochondria-targeted antioxidant mitigates injury. Am. J. Physiol. Renal Physiol..

[3] Li S., Wu H., Han D., Ma S., Fan W., Wang Y., Zhang R., Fan M., Huang Y., Fu X. (2018). A Novel Mechanism of Mesenchymal Stromal Cell-Mediated Protection against Sepsis: Restricting Inflammasome Activation in Macrophages by Increasing Mitophagy and Decreasing Mitochondrial ROS. Oxid. Med. Cell. Longev..

[4] Arulkumaran N., Pollen S.J., Tidswell R., Gaupp C., Peters V.B.M., Stanzani G., Snow T.A.C., Duchen M.R., Singer M. (2021). Selective mitochondrial antioxidant MitoTEMPO reduces renal dysfunction and systemic inflammation in experimental sepsis in rats. Br. J. Anaesth..

[5] Song Y., Xing H., He Y., Zhang Z., Shi G., Wu S., Liu Y., Harrington E.O., Sellke F.W., Feng J. (2021). Inhibition of mitochondrial reactive oxygen species improves coronary endothelial function after cardioplegic hypoxia/reoxygenation. J. Thorac. Cardiovasc. Surg..

[6] Weidinger A., Müllebner A., Paier-Pourani J., Banerjee A., Miller I., Lauterböck L., Duvigneau J.C., Skulachev V.P., Redl H., Kozlov A.V. (2015). Vicious Inducible Nitric Oxide Synthase-Mitochondrial Reactive Oxygen Species Cycle Accelerates Inflammatory Response and Causes Liver Injury in Rats. Antioxid. Redox Signal..

[7] Rademann P., Weidinger A., Drechsler S., Meszaros A., Zipperle J., Jafarmadar M., Dumitrescu S., Hacobian A., Ungelenk L., Röstel F. (2017). Mitochondria-targeted antioxidants SkQ1 and MitoTEMPO failed to exert a long-term beneficial effect in murine polymicrobial sepsis. Oxid. Med. Cell. Longev..

[8] Powell R.D., Goodenow D.A., Britton Christmas A., McKillop I.H., Evans S.L. (2018). Effect of systemic triphenylphosphonium on organ function and oxidative stress. Am. Surg..

[9] Ozsvari B., Sotgia F., Lisanti M.P. (2018). Exploiting mitochondrial targeting signal(s), TPP and bis-TPP, for eradicating cancer stem cells (CSCs). Aging.

[10] Trnka J., Elkalaf M., Andě M. (2015). Lipophilic triphenylphosphonium cations inhibit mitochondrial electron transport chain and induce mitochondrial proton leak. PLoS ONE.

[11] Cadenas S. (2018). Mitochondrial uncoupling, ROS generation and cardioprotection. Biochim. Biophys. Acta -Bioenerg..

[12] Weidinger A., Kozlov A.V. (2015). Biological Activities of Reactive Oxygen and Nitrogen Species: Oxidative Stress versus Signal Transduction. Biomolecules.

[13] Speer C.P. (2001). New insights into the pathogenesis of pulmonary inflammation in preterm infants. Biol. Neonate.

[14] Brooks D., Barr L.C., Wiscombe S., McAuley D.F., Simpson A.J., Rostron A.J. (2020). Human lipopolysaccharide models provide mechanistic and therapeutic insights into systemic and pulmonary inflammation. Eur. Respir. J..

[15] Zuo L., Otenbaker N.P., Rose B.A., Salisbury K.S. (2013). Molecular mechanisms of reactive oxygen species-related pulmonary inflammation and asthma. Mol. Immunol..

[16] Kratzer E., Tian Y., Sarich N., Wu T., Meliton A., Leff A., Birukova A.A. (2012). Oxidative stress contributes to lung injury and barrier dysfunction via microtubule destabilization. Am. J. Respir. Cell Mol. Biol..

[17] Gandhirajan R.K., Meng S., Chandramoorthy H.C., Mallilankaraman K., Mancarella S., Gao H., Razmpour R., Yang X.-F., Houser S.R., Chen J. (2013). Blockade of NOX2 and STIM1 signaling limits lipopolysaccharide-induced vascular inflammation. J. Clin. Investig..

[18] Fu P., Epshtein Y., Ramchandran R., Mascarenhas J.B., Cress A.E., Jacobson J., Garcia J.G.N., Natarajan V. (2021). Essential role for paxillin tyrosine phosphorylation in LPS-induced mitochondrial fission, ROS generation and lung endothelial barrier loss. Sci. Rep..

[19] Song J.-A., Yang H.-S., Lee J., Kwon S., Jung K.J., Heo J.-D., Cho K.-H., Song C.W., Lee K. (2010). Standardization of bronchoalveolar lavage method based on suction frequency number and lavage fraction number using rats. Toxicol. Res..

[20] Ray A., Dittel B.N. (2010). Isolation of mouse peritoneal cavity cells. J. Vis. Exp..

[21] Zhou M., Diwu Z., Panchuk-Voloshina N., Haugland R.P. (1997). A stable nonfluorescent derivative of resorufin for the fluorometric determination of trace hydrogen peroxide: Applications in detecting the activity of phagocyte NADPH oxidase and other oxidases. Anal. Biochem..

[22] Jais A., Einwallner E., Sharif O., Gossens K., Lu T.T.-H., Soyal S.M., Medgyesi D., Neureiter D., Paier-Pourani J., Dalgaard K. (2014). Heme oxygenase-1 drives metaflammation and insulin resistance in mouse and man. Cell.

[23] Weidinger A., Dungel P., Perlinger M., Singer K., Ghebes C., Duvigneau J.C., Müllebner A., Schäfer U., Redl H., Kozlov A.V. (2013). Experimental data suggesting that inflammation mediated rat liver mitochondrial dysfunction results from secondary hypoxia rather than from direct effects of inflammatory mediators. Front. Physiol..

[24] Müllebner A., Moldzio R., Redl H., Kozlov A.V., Duvigneau J.C. (2015). Heme Degradation by Heme Oxygenase Protects Mitochondria but Induces ER Stress via Formed Bilirubin. Biomolecules.

[25] Sommer N., Hüttemann M., Pak O., Scheibe S., Knoepp F., Sinkler C., Malczyk M., Gierhardt M., Esfandiary A., Kraut S. (2017). Mitochondrial Complex IV Subunit 4 Isoform 2 Is Essential for Acute Pulmonary Oxygen Sensing. Circ. Res..

[26] Park W.H. (2021). Tempol differently affects cellular redox changes and antioxidant enzymes in various lung-related cells. Sci. Rep..

[27] Jirillo E., Caccavo D., Magrone T., Piccigallo E., Amati L., Lembo A., Kalis C., Gumenscheimer M. (2002). The role of the liver in the response to LPS: Experimental and clinical findings. J. Endotoxin Res..

[28] Wideman R.F., Bowen O.T., Erf G.F. (2009). Broiler pulmonary hypertensive responses during lipopolysaccharide-induced tolerance and cyclooxygenase inhibition. Poult. Sci..

[29] Sheng S.-J., Nie Y.-C., Lin F., Li P.-B., Liu M.-H., Xie C.-S., Long C.-F., Su W.-W. (2014). Biphasic modulation of α-ENaC expression by lipopolysaccharide in vitro and in vivo. Mol. Med. Rep..

[30] Dauphinee S.M., Karsan A. (2006). Lipopolysaccharide signaling in endothelial cells. Lab. Investig..

[31] López-Ongil S., Senchak V., Saura M., Zaragoza C., Ames M., Ballermann B., Rodríguez-Puyol M., Rodríguez-Puyol D., Lowenstein C.J. (2000). Superoxide regulation of endothelin-converting enzyme. J. Biol. Chem..

[32] Pak O., Scheibe S., Esfandiary A., Gierhardt M., Sydykov A., Logan A. (2018). Impact of the mitochondria-targeted antioxidant MitoQ on hypoxia-induced pulmonary hypertension. Eur. Respir. J..

[33] Wynne B.M., Zou L., Linck V., Hoover R.S., Ma H.-P., Eaton D.C. (2017). Regulation of Lung Epithelial Sodium Channels by Cytokines and Chemokines. Front. Immunol..

[34] Mittal M., Siddiqui M.R., Tran K., Reddy S.P., Malik A.B. (2014). Reactive oxygen species in inflammation and tissue injury. Antioxid. Redox Signal..

[35] Burghuber O., Mathias M.M., McMurtry I.F., Reeves J.T., Voelkel N.F. (1984). Lung edema due to hydrogen peroxide is independent of cyclooxygenase products. J. Appl. Physiol..

[36] Tzeng H.-P., Ho F.-M., Chao K.-F., Kuo M.-L., Lin-Shiau S.-Y., Liu S.-H. (2003). beta-Lapachone reduces endotoxin-induced macrophage activation and lung edema and mortality. Am. J. Respir. Crit. Care Med..

[37] Nakamura M., Motoyama S., Saito S., Minamiya Y., Saito R., Ogawa J. (2004). Hydrogen peroxide derived from intestine through the mesenteric lymph induces lung edema after surgical stress. Shock.

[38] Mkrtchyan G.V., Üçal M., Müllebner A., Dumitrescu S., Kames M., Moldzio R., Molcanyi M., Schaefer S., Weidinger A., Schaefer U. (2018). Thiamine preserves mitochondrial function in a rat model of traumatic brain injury, preventing inactivation of the 2-oxoglutarate dehydrogenase complex. Biochim. Biophys. Acta. Bioenerg..

