# Supplementary material for: Systemic Effects of mitoTEMPO upon Lipopolysaccharide Challenge Are Due to Its Antioxidant Part, While Local Effects in the Lung Are Due to Triphenylphosphonium
Source: Antioxidants (Basel). 2022 Feb 6;11(2):323. doi: 10.3390/antiox11020323 (PMC8868379; doi:10.3390/antiox11020323)
Supplement: Supplementary file 1 [file antioxidants-11-00323-s001.zip › antioxidants-1557353-supplementary material.pdf]

Supplementary Materials S1

Preparation of lung tissue slices

Precision cut lung slices (PCLS) were prepared from freshly harvested Sprague Dawley rat lungs. After euthanasia and dissection of the animal, a small cut was made in the trachea, and the lung was injected with 3 - 5 mL of 2 % agarose in distilled water solution (38 °C) with a syringe, followed by putting the lung on ice for quick gelation of the agarose. This technique inflates the collapsed lung, restoring morphological structures while preserving the viability of the tissue. Additionally, agarose gel contributes to the overall structural strength of the sample, making it suitable for PCLS preparation. Cylindrical tissue cores of 8 mm were punched, and slices of 200 μm thickness were cut using a microtome (Compresstome® VF-300, Precisionary Instruments, NC, US).

Experiment 1

The slices were stained at room temperature in a carbonate-free buffer (CFB; 140 mM NaCl, 3 mM KCl, 2 mM $CaCl_2$, 2 mM $MgCl_2$, 10 mM HEPES, and 20 mM Glucose, pH 7.4) on a shaker (60 revolutions per minute) covered with aluminium foil to prevent light exposure. H2DCFDA (10 μM, ex/em 488/520 nm; Thermo Fischer, MA, USA) was used to stain cytoplasmic ROS, and NucSpot® Live 650 (1x; ex/em 633/675 nm; Biotium Inc., CA, USA) to stain nuclei. After 60 min, the medium was replaced with 250 μL pre-warmed fresh CFB, and the slices were held in place with a hollow metallic cylinder covered with thin nylon net. The samples were inspected under a confocal laser scanning microscope (LSM 510, Zeiss, Germany) to adjust the image acquisition settings. Afterwards, the treatments (diluted in 100 μL of CFB) were accurately pipetted in the wells, resulting in the following final concentrations (Supplementary Table S1). After the addition of the treatments, the images were recorded by using different positions and time series program, representing the molecular kinetics at 60 min.

Experiments 2

The slices were stained, treated, and incubated at room temperature in CFB on a shaker (60 revolutions per minute) covered with aluminium foil to prevent light exposure. The treatments (Supplementary Table S1) and the fluorescent dyes H2DCFDA (10 μM) and NucSpot® Live 650 (1x) were added simultaneously to the PCLS and incubated for 60 min. Then, the medium was replaced with 250 μL pre-warmed fresh CFB and the slices were held in place with a hollow metallic cylinder covered with thin nylon net. Afterwards, the samples were inspected under a confocal laser scanning microscope (LSM 510, Zeiss, Germany) to adjust the image acquisition settings. The images were recorded by using different positions and time series program, representing the molecular kinetics at 60 min.

Experiment no.	ctrl	LPS	LPS +TPP	LPS + mitoTEMPO
1	-	6 μg/mL	6 μg/mL + 10 μM	6 μg/mL + 10 μM
2	-	60 μg/mL	60 μg/mL + 30 μM	60 μg/mL + 30 μM

Supplementary Table S1. Final concentrations of PCLS treatment. LPS=lipopolysaccharide; TPP=triphenylphosphonium

Supplementary Data

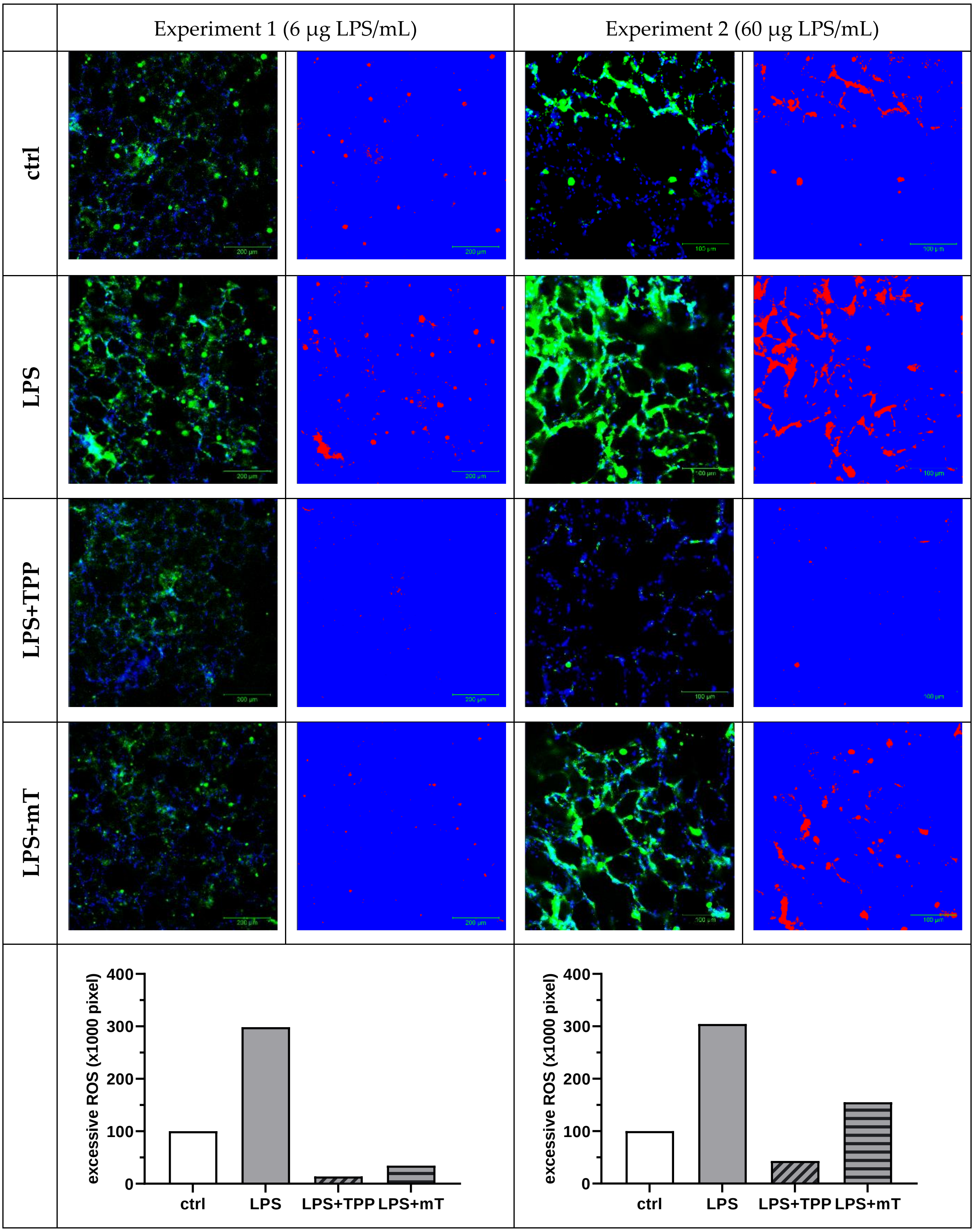

Supplementary Figure S1. LSM images of precision cut lung slices. Green signal represents cytoplasmic ROS (10 µM H2DCFDA), and blue signal represents counterstained nuclei (1x NucSpot® Live 650). The images in the left column of experiment 1 and 2 reveal the morphological structure of the lung tissue by exposing the nuclei of the cells together with distribution of the intracellular ROS. The images in the right column of experiment 1 and 2 represent the same tissue, however, the red-coloured area shows how large are the areas with ROS overproduction (overproduction was identified if the intensity of fluorescence was above a threshold of 254 units out of 256 representing the upper limit of the sensitivity range). The panels in the bottom represent the quantitative evaluation of areas with excessive ROS production for experiment 1 and 2, respectively. Scale bar experiment 1 = 200 µm, experiment 2 = 100 µm; LPS=lipopolysaccharide; TPP=methyltriphenylphosphonium chloride; mT=mitoTEMPO
